# Ursolic acid reverses liver fibrosis by inhibiting NOX4/NLRP3 inflammasome pathways and bacterial dysbiosis

**DOI:** 10.1080/19490976.2021.1972746

**Published:** 2021-09-16

**Authors:** Yuan Nie, Qi Liu, Wang Zhang, Yipeng Wan, Chenkai Huang, Xuan Zhu

**Affiliations:** Department of Gastroenterology, The First Affiliated Hospital of Nanchang University, Nanchang, Jiangxi, China

**Keywords:** Liver fibrosis, NADPH oxidase 4, NLRP3 inflammasome, ursolic acid, intestinal bacteria

## Abstract

Activation of the NOX4/NLRP3 inflammasome pathway has been associated with fibrosis in other organs. An imbalance in intestinal bacteria is an important driving factor of liver fibrosis through the liver-gut axis. This study aimed to explore whether the effect of ursolic acid (UA) on liver fibrosis was associated with the NOX4/NLRP3 inflammasome pathways and intestinal bacteria. Wild-type (WT), *NLRP3^−/-^*, and *NOX4^−/-^* mice and AP-treated mice were injected with CCI4 and treated with or without UA. The intestinal contents of the mice were collected and analyzed by 16S rRNA sequencing. UA alleviated liver fibrosis, which manifested as decreases in collagen deposition, liver injury, and the expression of fibrosis-related factors, and the expression of NOX4 and NLRP3 was significantly inhibited by UA treatment. Even after CCI4 injection, liver damage and fibrosis-related factors were significantly decreased in *NLRP3^−/-^, NOX4^−/-^*, and AP-treated mice. Importantly, the expression of NLRP3 was obviously inhibited in *NOX4^−/-^* and AP-treated mice. In addition, the diversity of intestinal bacteria and the abundance of probiotics in *NLRP3^−/-^* and *NOX4^−/-^* mice was significantly higher than those in WT mice, while the abundance of harmful bacteria in *NLRP3^−/-^* and *NOX4^−/-^* mice was significantly lower than that in WT mice. The NOX4/NLRP3 inflammasome pathway plays a crucial role in liver fibrosis and is closely associated with the beneficial effect of UA. The mechanism by which the NOX4/NLRP3 inflammasome pathway is involved in liver fibrosis may be associated with disordered intestinal bacteria.

## Introduction

A key factor of liver fibrosis is the activation of hepatic stellate cells (HSCs), which differentiate into activated myofibroblasts.^1^ Uncontrolled chronic damage will lead to liver cirrhosis, which has high morbidity and mortality.^2^ Therefore, studying the pathogenesis of liver fibrosis can lay the foundation for future treatments and is essential in delaying the development of liver fibrosis.

NADPH oxidase 4 (NOX4) is the most widely distributed NOX catalytic subunit.^3,4^ NOX4 is abundantly present in hepatocytes and HSCs and can directly or indirectly regulate the cell signaling network through reactive oxygen species (ROS).^3^ NOX4 is the key factor that promotes liver fibrosis via apoptosis and HSCs activation. NLRP3 is an intracellular multiprotein complex that is widely involved in cellular immunity and can respond to harmful signals (pathogen-associated molecular patterns (PAMPs) and damage-associated molecular patterns (DAMPs), thereby activating caspase1 through autocatalysis, releasing proinflammatory cytokines (such as IL-1β and IL-18), and initiating the repair response.^5,6^ Previous studies showed that NLRP3 inflammasome activation is associated with ovarian,^7^ kidney^8^ and heart^9^ fibrosis. However, the role of the NOX4/NLRP3 signaling pathway in liver fibrosis has rarely been studied.

The intestine and liver communicate closely through the biliary tract, portal vein and systemic circulation. A common feature of liver cirrhosis is an increase in potentially pathogenic bacteria and a decrease in beneficial bacteria.^10^ The imbalance in intestinal microbes will cause the bacterial translocation and their products, lead to an inflammatory cascade and result in liver damage and even liver fibrosis.^11^ Once liver cirrhosis worsens, it will lead to intestinal barrier dysfunction and pathological bacterial translocation.^12^ The occurrence and progression of liver fibrosis is closely associated with intestinal bacterial disorders. Previous research has shown that the NLRP3 inflammasome can recognize intestinal bacteria and their metabolites, exert intestinal immune functions and thus protect the intestinal tract.^13^

Ursolic acid (UA) is a natural compound that is widely found in a variety of plants and has been shown to have antifibrotic effects.^14,15^ UA alleviates liver fibrosis by inhibiting liver inflammation, selectively inducing apoptosis in activated HSCs and inhibiting HSCs proliferation,^16^ but the specific mechanism is still unclear. Since NOX4 and the NLRP3 inflammasome are closely associated with the development and progression of fibrosis in many organs, these factors are also associated with changes in intestinal bacteria. We aimed to explore whether the antifibrosis mechanism of UA is associated with the NOX4/NLRP3 signaling pathway and the regulation of intestinal bacteria.

## Materials and methods

### Animal models and experimental design

The wild-type (WT) C57BL/6 mice used in this experiment were obtained from the Department of Laboratory Animal Science of Nanchang University, and both *NOX4^−/-^* mice and *NLRP3^−/-^* mice were purchased from the Jackson Laboratory in the United States. All animals were cared for humanely in accordance with the guidelines of the institution. The animals were kept in an environment with 12 hours of light and 12 hours of darkness, the room temperature was 20–24°C, the humidity was 50%-60%, and food and sterile water were freely available. In this study, CCI4 intraperitoneal injection and MCD (Methionine-choline deficient diet) diet were used to induce liver fibrosis. According to the principle of random allocation, WT mice weighing 20 ~ 30 g were divided into three groups (n = 10): the control group (injected intraperitoneally with 1 ml/kg olive oil twice per week for 8 weeks), CCI4 group (injected intraperitoneally with 1 ml/kg CCI4 twice per week for 8 weeks) and UA group (injected intraperitoneally with 1 ml/kg CCI4 twice per week for 8 weeks and administered 50 mg/kg/d UA by gavage for the last 4 weeks). According to the principle of random allocation, WT mice weighing 20 ~ 30 g were divided into three groups (n = 10): the MCS group (MCS feeding for 8 weeks), MCD group (MCD feeding for 8 weeks) and UA group (MCD feeding for 8 weeks and administered 50 mg/kg/d UA by gavage for the last 4 weeks). *NLRP3^−/-^* mice were randomly divided into the *NLRP3^−/-^* group (n = 10, same treatment as the CCI4 group) and the *NLRP3^−/-^*+UA group (n = 10, same treatment as the UA group). The *NOX4^−/-^* mice were randomly divided into 2 groups (n = 10): the *NOX4^−/-^* group (same treatment as the CCI4 group), the *NOX4^−/-^*+UA group (same treatment as the UA group). and the CCI4+ AP group (the mice were administered CCI4 for 4 weeks, and CCI4 plus 50 mg/kg/d AP (NOX4 biological inhibitor) was administered for an additional 4 weeks) were added. All experimental procedures were approved by the Animal Care and Use Committee of the First Affiliated Hospital of Nanchang University.

### Liver histopathology and immunohistochemistry

The mice were sacrificed by cervical dislocation, and whole liver and mouse fecal specimens (cecal contents) were collected. The liver and ileum were divided into two parts: one part was fixed in 4% paraformaldehyde for immunohistochemistry (IHC), while the other part was stored at −80°C for PCR and protein extraction experiments. The paraffin-embedded liver and ileum samples were used

to prepare 5 µm thick slices with a microtome. The liver samples were embedded in paraffin, sectioned and examined by hematoxylin and eosin (HE) staining, Masson’s trichrome staining, Sirius red staining, IHC, terminal deoxynucleotidyl transferase dUTP nick end labeling (TUNEL) and *E. coli* immunohistochemistry. For Sirius red collagen staining, the liver slices were deparaffinized and stained with Sirius Red for 1 h at room temperature. The degree of hepatic fibrosis was evaluated semiquantitative based on the Metavir score.

IHC was performed on serial sections of paraffin-embedded ileal tissue. After rehydrating, the sections were maintained in 0.3% H_2_O_2_ for 7 min to eliminate endogenous peroxidase and then were washed with phosphate buffer saline (PBS). Next, the samples were transferred to citrate buffer (pH 7.6) and heated in a microwave oven for 20 min. After washing the sections with PBS and blocking the nonspecific binding sites with 5% bovine serum albumin (BSA), they were incubated with rabbit anti-a-SMA (Abcam, ab5694), anti-Collagen-1 (Abcam, ab34710), anti-NOX4 (GeneTex, GTX31588), anti-NLRP3 (Abcam, ab263899), overnight at 4°C. Next, the sections were rinsed in PBS and then incubated with biotin-labeled goat anti-polyvalent for 15 min at 37°C and horseradish peroxidase-labeled streptavidin for 20 min at 37°C. The coloration was completed after treatment with diaminobenzidine for 10 min, after which the slides were counterstained with hematoxylin for 2 min, rinsed in tap water and dehydrated. Liver damage was assessed by two pathologists in a blinded manner.

Paraffin-fixed sections (4 μm thick) were prepared on slides. The sections were deparaffinized in xylene and rehydrated through a graded ethanol series. Endogenous peroxidase activity was blocked by incubation in a 3% H_2_O_2_ solution at room temperature for 8 min. Antigen retrieval was performed using boiling citrate buffer (pH 6.0) in a microwave for 15 min. The membranes were permeabilized by 0.3% triton-X100 for 15 min at 37°C and then blocked with 3% BSA for 60 min at room temperature. The sections were incubated with anti-Occludin (Abcam, ab167161), anti-Claudin-1 (Thermo Fisher Scientific, 2H10D10), anti-CD11b (Abcam, ab8878), anti-*Escherichia coli* (*E. coli*) (Abcam, ab20640) at 4°Covernight followed by incubation with Alexa Fluor-conjugated secondary antibody (1:500, Invitrogen) at 37°C for 30 min. DAPI was used to counterstain (Invitrogen) the nuclei. Fluorescence staining was observed under a normal fluorescence microscope.

### Quantitative real-time polymerase chain reaction (qRT-PCR)

Fluorescent chemicals were used to detect the total amount of products after each PCR cycle to analyze the relative abundance of the target gene. The primers used for qRT-PCR are shown in Online Supplementary Table S1. The qRT-PCR cycle parameters were as follows: initial denaturation at 95°C for 10 minutes, then denaturation at 95°C for 20 seconds, annealing at 60°C for 30 seconds, extension at 72°C for 30 seconds, and finally 72°C for 10 minutes. The ΔΔCT method was used to calculate the relative gene expression.

### 16S rRNA gene sequencing and analysis

The feces of the sacrificed mice were collected and analyzed by the 16S rRNA sequencing method to assess the composition of intestinal bacteria. Microbial DNA was extracted from intestinal contents using the E.Z.N.A.® soil DNA kit (Omega Biotek, USA) according to the manufacturer’s protocols. The final DNA concentration and purity were determined by a NanoDrop 2000 UV-vis spectrophotometer (Thermo Scientific, Wilmington, USA), and the DNA quality was checked by 1% agarose gel electrophoresis. The V3-V4 hypervariable regions of the bacterial 16S rRNA gene were amplified with the primers 338 F (5’-ACTCCTACGGGAGGCAGCAG-3’) and 806 R (5’-GGACTACHVGGGTWTCTAAT-3’) by a thermocycler PCR system (GeneAmp 9700, ABI, USA). The PCR components were as follows: 4 μL of 5 × Fast-Pfu buffer, 0.8 μL of 2.5 mmol/L deoxyribonucleotide triphosphates (dNTPs), 0.8 μL of each primer (5 μmol/L), 0.4 μL of Fast-Pfu polymerase, and 10 ng of template DNA. The PCR conditions were 3 minutes of denaturation at 95°C, followed by 27 cycles of 30 seconds at 95°C, 30 seconds annealing at 55°C, 45 seconds elongation at 72°C, and finally 10 minutes extension at 72°C. The library was constructed according to the standard operating procedures of the Illumina MiSeq platform (Illumina, San Diego, USA). Sequencing was performed using the MiSeq PE300 Illumina platform.

The raw data were processed to obtain clean reads by eliminating adaptor pollution and low- quality sequences using Trimmomatic software, and then the reads were truncated at any site with an average quality score<20 over a 50 bp sliding window. After trimming, Fast Length Adjustment of Short reads (FLASH, v1.2.11) was used to combine tags with high-quality paired-end reads with an average read length of 252 bp. The effective reads were clustered as operational taxonomic units (OTUs) with a 97% similarity cutoff by the algorithm in USEARCH (version 7.1) software. The phylogenetic taxa of each 16S rRNA gene sequence was analyzed by the Ribosomal Database Project (RDP) Classifier (http://rdp.cme.msu.edu/) against the Silva (SSU128) 16S rRNA database using a confidence threshold of 70%.The feature sequence-level α-diversity index was used to evaluate the diversity of the sample, the β-diversity index was used to assess the differences in intestinal bacterial structure, and the results were then displayed by principal coordinates analysis (PCoA) and nonmetric multidimensional scaling (NMDS) maps. The differential abundance was compared using the Wilcoxon rank sum test. LEfSe was performed to identify the differential taxa between groups at the genus or higher taxonomy levels. The correlation between differential bacteria and clinical indicators was completed by Spearman Correlation Coefficient. The image visualization was completed by heatmap package, VEGAN package, ggplot2 package of R language.

### Measurement of indices by enzyme-linked immunosorbent assay (ELISA)

After 8 weeks, all mice were fasted for 72 hours and anesthetized with ether and blood was collected in a 1.5 ml sterile EP tube. The blood was allowed to stand for 10 minutes and was centrifuged for 10 minutes (4°C, 3000 rpm/min) after clotting. The supernatant was collected and sent to the Laboratory Department of the First Affiliated Hospital of Nanchang University. The alanine aminotransferase (ALT), aspartate aminotransferase (AST), triglycerides and hydroxyproline levels in mice serum were determined using ELISA kits from Shanghai Tongwei Industry Co., Ltd. (Shanghai, China) according to the manufacturer’s instructions. The gut barrier index, as measured by lipopolysaccharide (LPS) and D-lactate (D-lac) levels, was determined using a commercial kit (Abcam, UK).

### Statistical analysis

Quantitative data are expressed as the means ± standard deviation (SD). Normality was assessed using the Kolmogorov-Smirnov test, and normally distributed data were analyzed by one-way analysis of variance (ANOVA). Statistical analyses were performed with IBM SPSS statistics version 26.0. GraphPad Prism 8.0 was used for image production. Values of *P* < .05 were considered significant.

## Results

### UA reverses liver damage, hepatic fibrosis and bacterial translocation

**Figure 1.**
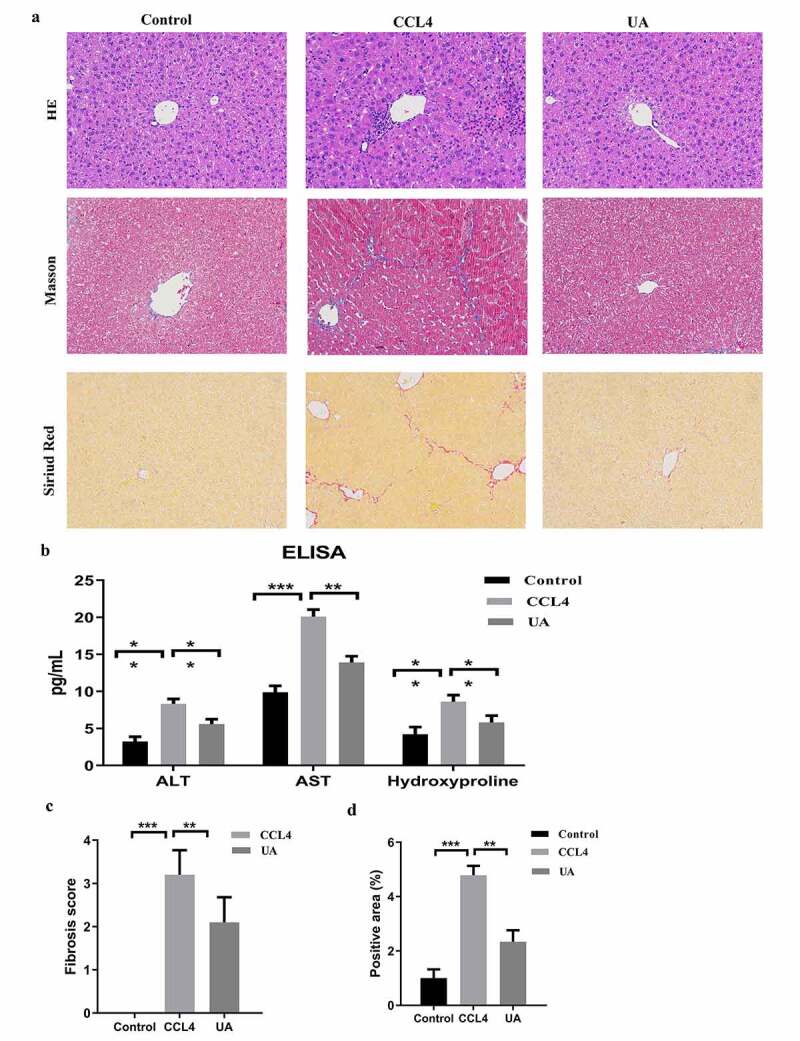
UA ameliorates liver damage and hepatic fibrosis in mice with CCI4-induced liver fibrosis. (a): HE staining, Masson’s trichrome staining and Sirius red staining (200× magnification); (b): The levels of ALT, AST and hydroxyproline were measured by ELISA; (c): The degree of fibrosis was assessed by fibrosis scoring and the positive area (%). n = 6 per group. The data are presented as the means±SD in each group. *P < .050, **P < .010 and ***P < .001

HE staining, Masson’s trichrome staining and Sirius red staining were used to observe the effect of UA on liver fibrosis. UA treatment significantly reduced the effects of CCI4 on the structure of liver lobules, fibrous septum formation and collagen deposition in mice, with reduced inflammatory cell infiltration (*P* < .010) (Figure 1a,c,d and Supplementary Figure S1). To determine the effect of UA on liver function, we evaluated the serum levels of ALT and AST and measured the liver levels of hydroxyproline, which is the main component of collagen tissue (Figure 1b). The levels of ALT, AST and hydroxyproline in the CCI4 group were significantly improved (*P* < .050) compared with those in the control group (*P* < .050). However, this effect was decreased by UA treatment. Similar results can be seen in MCD induced liver fibrosis model (Supplementary Figure S2).

**Figure 2.**
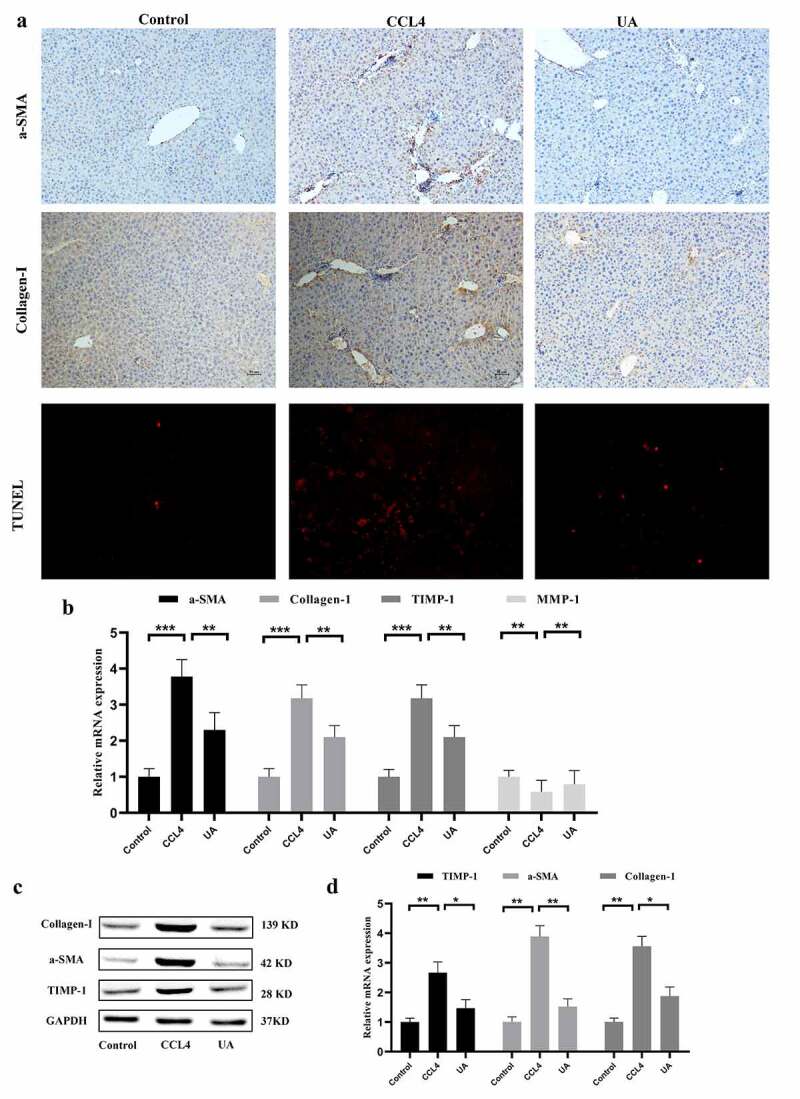
UA exerts antifibrotic effects by impacting the expression of fibrosis-related factors. (a): IHC was used to measure the levels of α-SMA and Collagen-1. Hepatocyte apoptosis was assessed by TUNEL assays; (b): The expression of α-SMA, Collagen-1, TIMP-1 and MMP-1 was determined by qRT-PCR; (c,d): The levels of Collagen-1, TIMP-1 and MMP-1 were determined by Western blotting. n = 6 per group. The data are presented as the means±SD in each group. *P < .050, **P < .010 and ***P < .001

Liver fibrosis is often accompanied by changes in the expression of fibrosis-related factors. IHC showed that the biomarkers of activated HSCs, including α-SMA and Collagen-1,^17,18^ in the liver tissues of the CCI4 group were significantly higher than those of the control group (*P* < .050). The TUNEL assay results showed that hepatocyte apoptosis in the CCI4 group was increased. However, in the UA group, these two effects were significantly reversed (Figure 2a). This result suggested that UA could inhibit HSCs activation and hepatocyte apoptosis. At the mRNA and protein levels, the expression of liver fibrosis-related factors (α-SMA, Collagen-1, and TIMP-1^17^) increased in the CCI4 group (*P* < .001), while the expression of the antifibrotic factor MMP-1 decreased^19^ (*P* < .010). After UA treatment, the expression of the antifibrotic factor MMP-1 increased (*P* < .010) (Figure 2b–d). This result suggests that UA can reverse liver fibrosis.

As we all know that LPS is a component of bacterial cell wall, so LPS in serum is often used to reflect the. The gut barrier index, as measured by LPS and D-lac levels, was determined using ELISA. As shown in Supplementary Figure S3, the level of LPS and D-lac of CCI4 groups were significantly higher than control groups. And the level of LPS and D-lac were significantly decreased in UA treatment groups (*P* < .050). In order to better show the bacterial translocation, *E. coli* immunofluorescence of liver tissue were conducted. The signal intensity of *E. coli* in CCI4 group were higher than control group, however, the increasing trend were inhibited in UA treatment group (Supplementary Figure 3A).

Mechanical barrier is the most important component of intestinal mucosal barrier, which is based on the integrity of intestinal epithelial cells and the tight junction between epithelial cells. When intestinal inflammation occurs, the intestinal mucosal barrier is damaged and the permeability is increased, so that the bacteria in the intestinal tract can transfer through the damaged intestinal mucosa. The expression of tight junction protein (Claudin-1 and Occludin) of ileum tissue were measured by immunofluorescence, qRT-PCR. As shown in Supplementary Figure S4, the level of Claudin-1 and Occludin of CCI4 groups were significantly lower than control groups. And the level of Claudin-1 and Occludin were significantly increased in UA treatment groups (*P* < .050).

### UA reduces liver fibrosis by inhibiting NOX4 and NLRP3 inflammasome signaling

**Figure 3.**
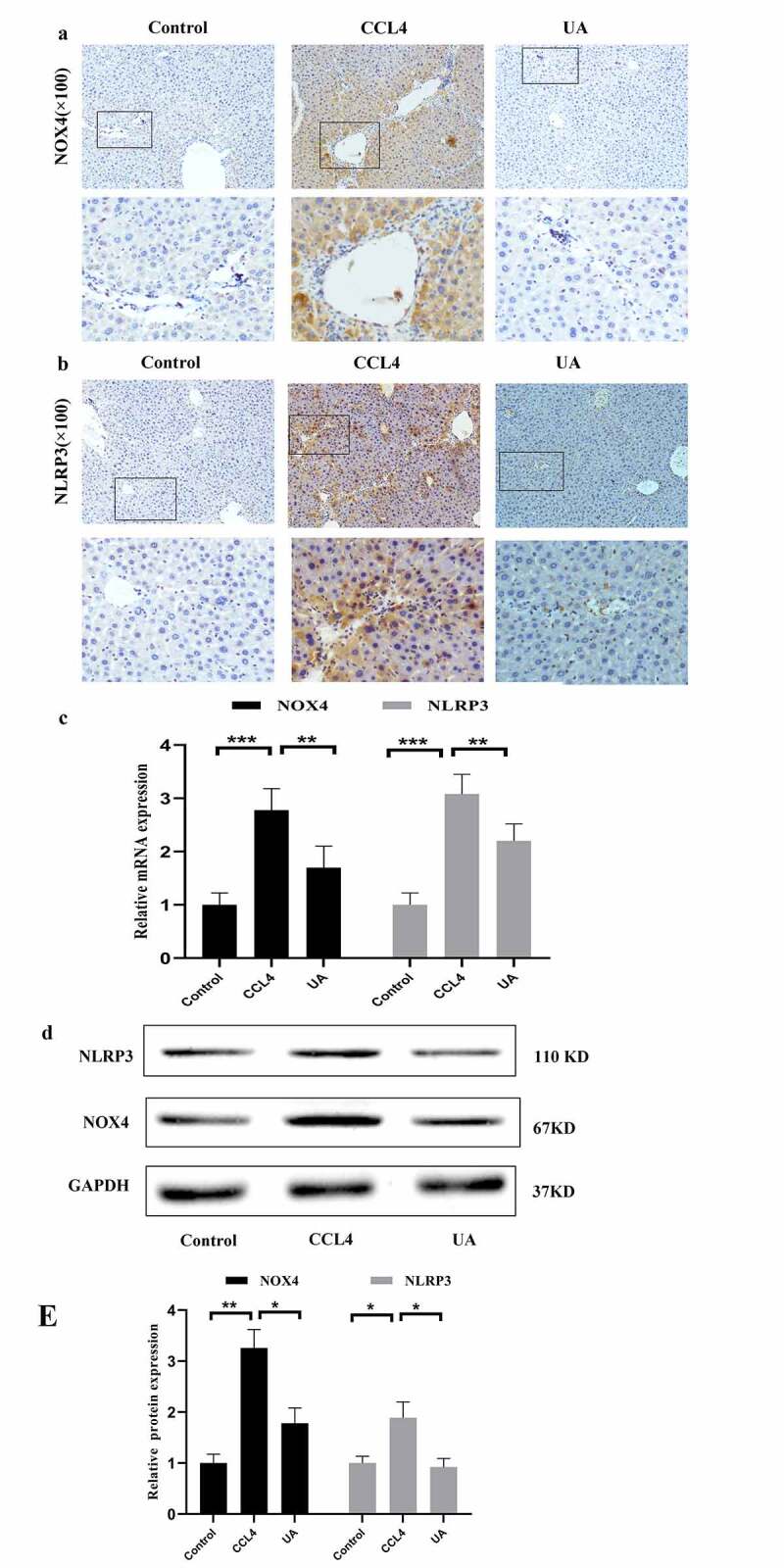
UA reduces CCI4-induced liver fibrosis by inhibiting NOX4 and NLRP3 signaling. (a) IHC was used to examine the effect of UA on NOX4 (100×); (b) IHC was used to examine the effect of UA on NLRP3 (100×); (c) The relative mRNA expression of NOX4 and NLRP3 was measured by qRT-PCR. n = 6 per group. The data are presented as the means±SD in each group. *P < .050, **P < .010 and ***P < .001

To explore the role of the NOX4/NLRP3 inflammasome pathway in liver fibrosis, we measured the expression of NOX4/NLRP3 in the three groups of mice. The IHC results showed that the expression of NOX4 and NLRP3 was upregulated in mice with CCI4 induced liver fibrosis, and after UA treatment, the expression of NOX4 and NLRP3 was significantly reduced (Figure 3a) (*P* < .050). PCR and Western blotting (*P* < .050) showed similar results (Figure 3c–e) (*P* < .010). This finding demonstrated that the development of liver fibrosis was associated with the expression of these two factors.

### Inhibiting NLRP3 expression can effectively alleviate liver fibrosis

**Figure 4.**
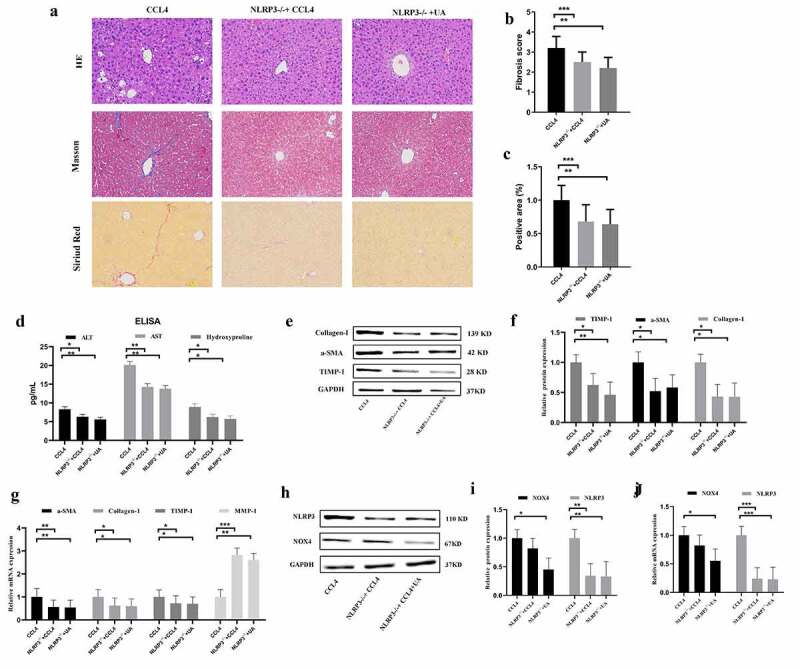
Effect of UA on the expression of NLRP3 inflammasomes in *NLRP3^−/-^* mice. (a): HE staining, Masson’s trichrome staining and Sirius red staining (200× magnification); (b,c) The degree of fibrosis was assessed by fibrosis scoring and the positive area (%); (d) The levels of ALT, AST and hydroxyproline were measured by ELISA; (e-f) The expression of Collagen-1, α-SMA and TIMP-1 was measured by Western blotting; (g) The expression of α-SMA, Collagen-1, TIMP-1 and MMP-1 was measured by qRT-PCR; (h-i) The protein levels of NOX4 and NLRP3 in *NLRP3^−/-^* mice with liver fibrosis were measured by Western blotting; (j) The mRNA levels of NOX4 and NLRP3 in *NLRP3^−/-^* mice with liver fibrosis were measured by qRT-PCR. n = 6 per group. The data are presented as the means±SD in each group. *P < .050, **P < .010 and ***P < .001

To further verify the relationship between NOX4 and NLRP3 inflammasome in the progression of liver fibrosis, *NOX4^−/-^* and *NLRP3^−/-^* mice were constructed. HE staining, Masson’s trichrome staining and Sirius red staining demonstrated that compared with those of WT mice injected with CCI4, collagen deposition was alleviated in the *NLRP3^−/-^* group (*P* < .001) (Figure 4a–c). In addition, the levels of ALT (*P* < .050), AST (*P* < .010) and hydroxyproline (*P* < .050) in CCI4 induced mice were significantly reduced after inhibiting NLRP3 (Figure 4d). The changes in the expression of liver fibrosis-related indicators were similar (Figure 4e–g), suggesting that NLRP3 is an important profibrotic factor in the process of liver fibrosis. Next, we explored whether NLRP3 was the target of the antifibrotic effect of UA. Compared with those in the *NLRP3^−/-^* group, the changes in collagen deposition and fibrous septum formation in the *NLRP3^−/-^*+UA group were not obvious. The changes in serum indexes and liver fibrosis-related factors were also not obvious (Figure 4a–g). In *NLRP3^−/-^*+UA mice, the mRNA and protein expression of NOX4 was decreased compared with that in the CCI4 group (*P* < .050) (Figure 4h–j).

### Inhibiting NOX4 expression can effectively alleviate liver fibrosis

**Figure 5.**
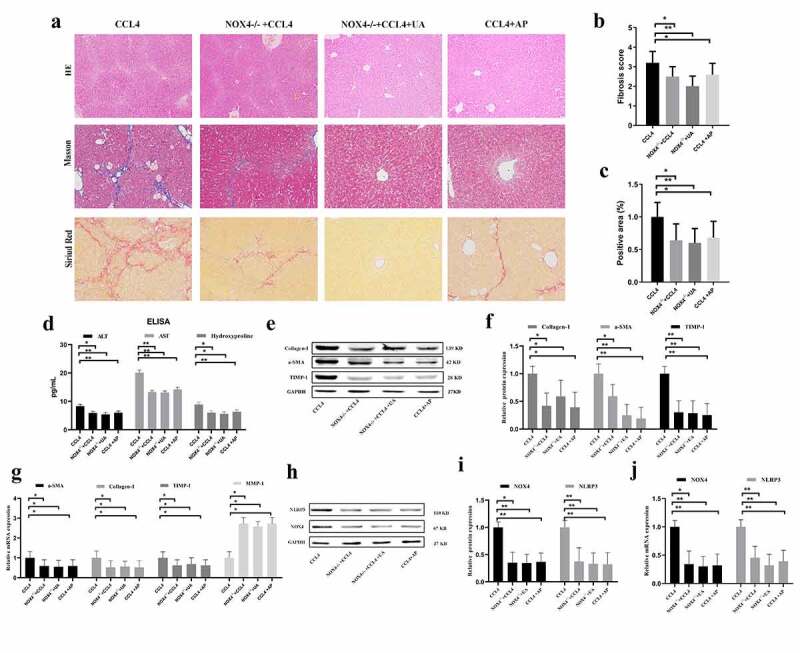
Effect of UA on the expression of NLRP3 in *NOX4^−/-^* mice. (a) HE staining, Masson’s trichrome staining and Sirius red staining (200× magnification); (b,c) The degree of fibrosis was measured by fibrosis scoring and the positive area (%); (d) The levels of ALT, AST and hydroxyproline were measured by ELISA; (e-f) The expression of Collagen-1, TIMP-1 and α-SMA was measured by Western blotting; (g) The expression of α-SMA, Collagen-1, TIMP-1 and MMP-1 was measured by qRT-PCR; (h–i) Liver protein levels of NOX4 and NLRP3 in *NOX4^−/^**^−^*** mice with liver fibrosis were measured by Western blotting; (j) The mRNA levels of NOX4 and NLRP3 in *NLRP3^−/-^* mice with liver fibrosis were measured by qRT-PCR. n = 6 per group. The data are presented as the means±SD in each group. *P < .050, **P < .010 and ***P < .001

We confirmed that UA exerts its antifibrotic effect by inhibiting NOX4. Compared with WT mice with liver fibrosis, mice in the *NOX4^−/-^*+CCI4 and CCI4+ AP groups showed lower collagen deposition (*P* < .050) (Figure 5a–c). Similarly, the levels of ALT, AST and hydroxyproline were lower than those in WT mice (Figure 5d), indicating that liver function was significantly restored by inhibiting NOX4. The expression of related fibrotic factors also indicates that NOX4 plays an important role in the development of liver fibrosis (Figure 5e–g). These results showed that inhibiting NOX4 could improve liver fibrosis and that NOX4 was an important profibrotic factor. Next, we assessed whether UA could reverse liver fibrosis by suppressing NOX4-related signals. HE staining, Masson’s trichrome staining and Sirius red staining indicated that compared with those of the *NOX4^−/-^*+CCI4 group and the CCI4+ AP group, there were no significant differences in collagen deposition and fibrous septum formation in the *NOX4^−/-^*+UA group, but the levels were still lower than those in the WT group (*P* < .010) (Figure 5a–c). Similarly, the serum indexes of liver function damage after UA treatment also recovered to a certain extent, the expression of profibrotic factors was also downregulated, and the expression of antifibrotic factors was upregulated (Figure 5d–g).

### NOX4 effectively stimulates NLRP3 expression

Our results confirmed that NOX4 and the NLRP3 inflammasome are two key molecules by which UA exerts antifibrotic effects. We further analyzed the upstream and downstream relationship between NOX4 and NLRP3. In *NLRP3*^−/-^+CCI4 mice, the mRNA and protein expression of NOX4 was not significantly decreased compared with that in WT+CCI4 mice (*P* > .050) (Figure 4h–j). Next, we determined the expression of NLRP3 in *NOX4^−/-^* mice. The mRNA and protein levels of NLRP3 in all *NOX4*^−/-^ mice were significantly lower than those in WT+CCI4 group mice (*P* < .010) (Figure 5h–j). These results suggested that NOX4 may be an upstream signal of NLRP3.

### Comparison of microbial abundance diversity

**Figure 6.**
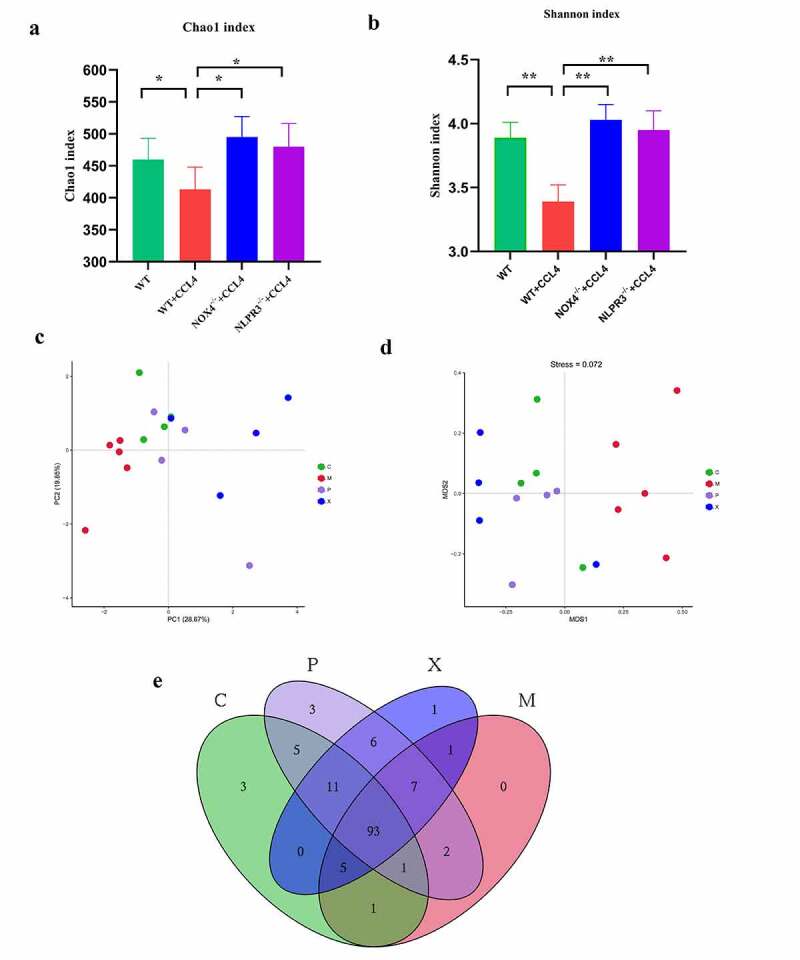
Changes in gut microbial diversity in four groups of mice with CCI4 induced liver fibrosis. (a,b):α-diversity analysis (Chao1 index, Shannon index) in the four groups;(c-d): β-diversity analysis (PCoA, NMDS) in the four groups; (e): Diversity of gut bacteria at the genus level in the four groups. Abbreviation C: control group; M: WT+CCI4 group; P: *NOX4^−/-^* +CCI4 group; X: *NLRP3^−/-^* +CCI4 group. n = 4, 4, 4 of C, P, X group; n = 5 of M group. The data are presented as the means±SD in four groups. *P < .050, **P < .010 and ***P < .001

The intestinal bacteria is an important indicator of intestinal and systemic states. Based on animal models of liver fibrosis, we explored the effect of intestinal bacteria during liver fibrosis and the effect of NOX4/NLRP3 inflammasome signal. The intestinal contents of mice were collected to assess the structure of intestinal bacteria by high-throughput sequencing. We examined the α-diversity indicators (Chao1 index, Shannon index) of the four groups to understand gut microbial diversity (Figure 6a,b) (*P* < .050). We observed that diversity in the CCI4 group was significantly lower than that in the control group, while diversity in the *NOX4^−/-^* group and the *NLRP3^−/-^* group were significantly improved compared with that in the CCI4 group. Next, we conducted β-diversity analysis to further evaluate the differences in intestinal structure between groups (Figure 6c,d). The PCoA plot and NMDS analysis showed differences in the intestinal structure in the control group, CCI4 group, *NOX4^−/-^* group and *NLRP3^−/-^* group, and the abundances of microbial species were also different (Figure 6e).

### Comparison of microbial abundance composition

**Figure 7.**
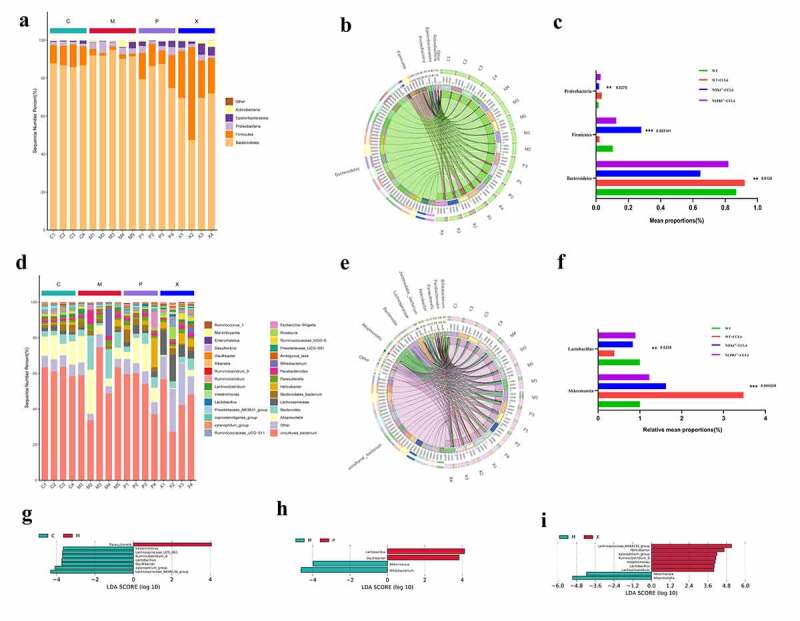
Microbial abundance in four groups of mice with CCI4 induced liver fibrosis. (a-c): Composition of microbial abundance at the phylum level in the four groups; (d-f): Composition of microbial abundance at species level in the four groups; (g-i): Different gut microbial abundances in the four groups are shown by the linear discriminant analysis (LDA) score (log_10_). Abbreviation C: control group; M: WT+CCI4 group; P: *NOX4^−/-^* +CCI4 group; X: *NLRP3^−/-^* +CCI4 group. n = 4, 4, 4 of C, P, X group; n = 5 of M group. The data are presented as the means±SD in four groups. *P < .050, **P < .010 and ***P < .001

In terms of microbial abundance, the performance of each group was different. At the gate level (Figure 7a–c), the abundance of the beneficial bacterium *Firmicutes* in the CCI4 group was lower than that in the control group (*P* < .001). *Firmicutes* abundance was significantly increased in the *NOX4^−/-^* group and *NLRP3^−/-^* group (*P* < .001). In contrast, the harmful bacterium *Proteobacteria* showed the opposite trend (*P* < .050). At the species level (Figure 7d–i), compared with the control group, mice in the CCI4 group carried more harmful *Akkermansia* bacteria (*P* < .001) and less beneficial *Lactobacillus* bacteria (*P* < .010) in their gut microbiota. Compared with the CCI4 group, the *NOX4^−/-^* group and the *NLRP3^−/-^* group had completely opposite trends.

### Relationship between the intestinal structure and environmental factors

**Figure 8.**
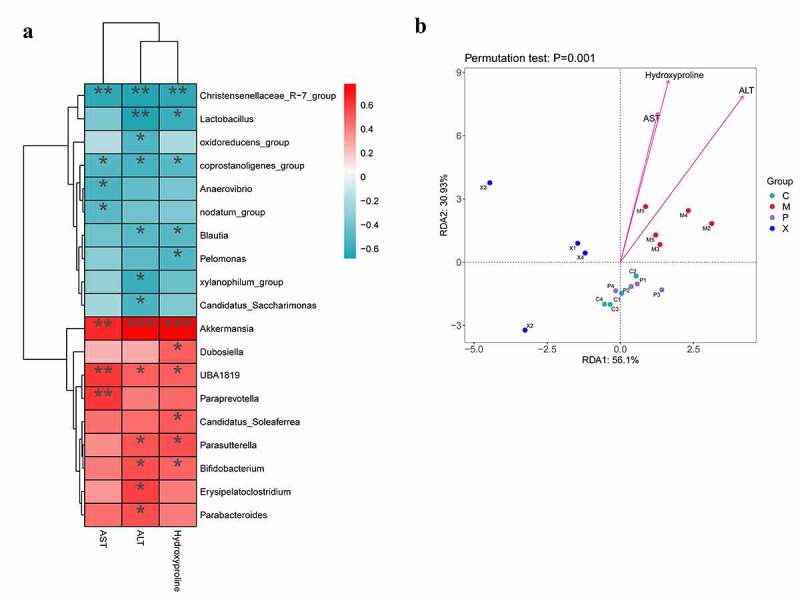
Potential associations between microbial abundance and AST, ALT, and hydroxyproline in mice with liver fibrosis. (a): The associations between microbial abundance and AST, ALT, and hydroxyproline are shown in heat maps; (b): Redundancy analysis (RDA) was performed to reveal the association of microbial abundance in relation to AST, ALT, and hydroxyproline based on the relative microbial abundance at different taxa levels using the R package “vegan”. Abbreviation C: control group; M: WT+CCI4 group; P: *NOX4^−/-^* +CCI4 group; X: *NLRP3^−/-^* +CCI4 group. n = 4, 4, 4 of C, P, X group; n = 5 of M group. The data are presented as the means±SD in four groups. *P < .050, **P < .010 and ***P < .001

The potential associations of intestinal structure with AST, ALT, and hydroxyproline were determined (Figure 8a,b). The beneficial bacteria *Lactobacillus* and the harmful bacteria *Akkermansia* was associated with liver function indicators (AST, ALT) and hydroxyproline, and the harmful bacteria *Akkermansia* was positively correlated with these three indicators. This result indicated that the abundance of harmful bacteria in the intestines increased with liver function damage. Inhibiting the NOX4/NLRP3 inflammasome signaling pathway could reduce the abundance of harmful bacteria and increase the abundance of beneficial bacteria. UA can improve the intestinal structure through the NOX4/NLRP3 inflammasome pathway.

## Discussion

In this study, we found that UA could alleviate CCI4 induced liver fibrosis, and proposed that the beneficial effect of UA on liver fibrosis may be achieved through the NOX4/NLRP3 inflammasome signaling pathway and improving intestinal bacteria.

HSCs are the main cell types that cause hepatic cirrhosis.^20^ In response to chronic injury, HSCs undergo activation, which is characterized by the loss of lipid droplets and the production of extracellular matrix (ECM) proteins.^2,21^ Activated HSCs and cross-linked ECM lead to liver fibrosis and subsequent liver failure.^2,22^ In the digestive system, liver cirrhosis is the most common nontumoral cause of death.^23^ Therefore, exploring the specific mechanism of liver fibrosis is critical for treating chronic liver disease. NOX has seven isoforms (NOX1-NOX5, DUOX1 and DUOX2), and these proteins are the main sources of ROS, while NOX4 plays an important role in the development of fibrosis.^24,25^ NOX4 is the most widely distributed NOX and can directly or indirectly regulate the cell signaling network through ROS. Previous research showed that NOX4 is the key to liver cell apoptosis and HSCs activation in CCI4 induced WT mice.^26–28^ We further demonstrated the role of NOX4 in *NOX4^−/-^* mice with liver fibrosis. Our study showed that NOX4 expression in mice with liver fibrosis was increased, while the degree of fibrosis in *NOX4^−/-^* mice was reduced. This finding indicates that NOX4 is involved in liver fibrosis. The relationship between the NLRP3 inflammasome and fibrosis has been reported in various studies. The NLRP3 inflammasome can be activated by high androgen levels, leading to ovarian interstitial cell fibrosis;^7^ NLRP3 inflammasome activation is associated with the development of renal fibrosis in diabetic nephropathy;^8^ and NLRP3/IL-1β activation is associated with the formation of cardiac fibrosis.^29^ However, the relationship between the NLRP3 inflammasome and liver fibrosis has rarely been reported. In our study, CCI4 was injected into WT mice and *NLRP3^−/-^* mice to establish liver fibrosis models, and the results showed that the expression of the NLRP3 inflammasome increased in mice with liver fibrosis. *NLRP3^−/-^* mice exhibited decreased in inflammatory cell infiltration and reduced liver fibrosis. In addition, the serum indicators of liver function in mice and liver fibrosis-related factors were also decreased. These results indicated that the NLRP3 inflammasome plays an important role in liver fibrosis and that inhibiting NLRP3 inflammasome expression can protect the liver.

Current studies have shown that NOX4 and NLRP3 can induce a variety of diseases have upstream and downstream regulatory relationships. In metabolic diseases, NOX4-dependent fatty acid oxidation promotes the activation of NLRP3 inflammasomes in macrophages and leads to the emergence of diseases, and the NOX4 inhibitor GKT137831 can inhibit the NLRP3 inflammasome.^30^ In diabetic rats, the protective effect of acarbose on vascular endothelial function was achieved by inhibiting the NOX4/NLRP3 inflammasome pathway.^31^ In acute kidney injury induced by lipopolysaccharide, dexmedetomidine inhibited NLRP3 activation by regulating the TLR4/NOX4/NF-κB pathway, thereby reducing disease development.^32^ However, the mutual regulation of NOX4 and NLRP3 in animal models of liver fibrosis is unknown. Therefore, we generated *NLRP3^−/-^* and *NOX4^−/-^* mice with liver fibrosis. In *NLRP3^−/-^* mice, although the expression of NOX4 in the liver was reduced, the difference was not statistically significant, indicating that NLRP3 inhibition does not affect NOX4 expression. However, NLRP3 expression was significantly reduced in *NOX4^−/-^* mice. This finding demonstrated that NOX4 may be an upstream signal of NLRP3 in liver fibrosis and can reverse liver fibrosis by inhibiting the NOX4/NLRP3 inflammasome signaling pathway. However, a study of chronic granulomatosis showed that NLRP3 inflammasome activation does not depend on the ROS produced by NOX.^33^ Perhaps there are other signaling molecules that play indirect roles between NOX4 and NLRP3; thus, clarifying the relationship between NOX4 and NLRP3 inflammasomes in the development of liver fibrosis is very important for future treatment of liver diseases.

UA is a natural compound that is widely found in a variety of plants. UA inhibits HSC activation by inhibiting the activity and expression of NOX, thereby reversing liver fibrosis.^34^ Our experiments confirmed that UA could reduce collagen deposition by inhibiting NOX4. In *NLRP3^−/-^* mice, we showed for the first time that UA could reverse liver fibrosis by inhibiting the expression of the NLRP3 inflammasome. Our study demonstrated that UA exerted its unique effect on fibrosis by inhibiting the NOX4/NLRP3 inflammasome signaling pathway in vivo.

The connection between the intestine and the liver is achieved through the gut-liver axis. Changes in intestinal bacteria, which account for the majority of bacteria, cause destruction of the intestinal barrier, which can damage liver cells and result in reversible liver fibrosis.^35^ Studies have shown that rats or patients with liver cirrhosis exhibit pathological bacterial translocation and inflammatory reactions, and the disease progresses rapidly.^36^ It has also been reported that in patients with liver cirrhosis, the abundance of *Bacteroides* and *Firmicutes* is decreased, while the abundance of *Streptococcus, Veillonella* and *Enterobacteriaceae* (including *E. coli, Klebsiella, Proteus* and *Enterobacter*) is increased.^37–39^ After treatment with metronidazole combined with ciprofloxacin or neomycin, ampicillin, and vancomycin combined with metronidazole, the disease process was significantly slowed.^36^ A study showed that the broad-spectrum antibiotic rifaximin exerts its pharmacological effects by changing the function of *E. coli*.^40^ Increasing evidence shows that the progression of liver fibrosis is closely associated with disturbances in the intestinal bacteria.

Our study showed that the intestinal bacteria changed during liver fibrosis, and this change was closely associated with UA-mediated inhibition of the NOX4/NLRP3 inflammasome signaling pathway. At the phylum level, the abundance of the beneficial bacteria *Firmicutes* in mice with hepatic fibrosis was lower than that in the control group, and the microbial diversity was reduced. After knocking out NOX4 or the NLRP3 inflammasome, the bacterial diversity and abundance increased significantly. In contrast, the harmful bacteria *Proteobacteria* showed the opposite trend. At the species level, the harmful bacterium *Akkermansia* was more abundant in the intestinal microbiota of WT+CCI4 mice than in mice in the other groups, and the beneficial bacterium *Lactobacillus* was lower. This intestinal biological disorder was partially improved after key genes were knocked out. Our research confirmed that the body can affect the intestinal bacteria through the NOX4/NLRP3 inflammasome signaling pathway, and the abundance of intestinal bacteria correlates with liver function. There is a positive correlation between beneficial bacteria and liver function. Previous studies have reported the antibacterial effect of UA, but the mechanism by which UA improves intestinal bacteria is not clear. Our study showed that UA improved the intestinal bacteria by inhibiting the NOX4/NLRP3 inflammasome signaling pathway, thereby restoring liver function and slowing the occurrence and development of liver fibrosis. To further verify our results, we will evaluate the efficacy of UA at the clinical level. In short, the elaboration of this signaling pathway is helpful for both the diagnosis of chronic liver disease and the treatment of liver cirrhosis. There are also some limitations to the present study, such as the lack of results regarding the coculture of HSCs in vitro, and additional results regarding ROS intervention.

In summary, this study showed that the NOX4/NLRP3 inflammasome signaling pathway is essential in the development of liver fibrosis and an important target for the regulation of intestinal bacteria. UA exerts an antifibrotic effect through this signaling pathway, and NOX4/NLRP3 inflammasome constitutes a key antifibrotic target in the treatment of chronic liver disease.

## Supplementary Material

Supplemental Material

## Data Availability

The data used to support the findings of this study are available from the corresponding author upon request.

## Credit author statement

YN and QL contributed equally to this study. YN and QL designed and wrote the manuscript; YN experimental operation; WZ and YPW analyzed data. XZ and CKH critically revised the manuscript. All of the authors reviewed the manuscript and approved the final version.
