# Supplementary material for: Ursolic acid reverses liver fibrosis by inhibiting NOX4/NLRP3 inflammasome pathways and bacterial dysbiosis
Source: Gut Microbes. 2021 Sep 16;13(1):1972746. doi: 10.1080/19490976.2021.1972746 (PMC8451456; doi:10.1080/19490976.2021.1972746)
Supplement: Supplemental Material [file KGMI_A_1972746_SM9264.zip › Supplementary information/Supplementary table 1.docx]

Supplementary table 1: Primer sequences for RT-qPCR.

| Gene | Sequence |
| --- | --- |
| a-SMA | F: 5'-CGATAGAACACGGCATCATC-3' |
|  | R: 5'-CATCAGGCAGTTCGTAGCTC-3' |
| Collagen-1 | F: 5'-GGGGCAAGACAGTCATCGAA-3' |
|  | R: 5'-GGATGGAGGGAGTTTACACGAA-3' |
| TIMP-1 | F: 5'-CCACAGATATCCGGTTCGGCTACA-3' |
|  | R: 5'-GCACACCCCACAGCCAGCACTAT-3' |
| MMP-1 | F: 5'-GCTGATACTGACACTGGTACTG-3' |
|  | R: 5'-CAATCTTTTCTGGGAGCTG-3' |
| NOX4 | F: 5'-GGATCACAGAAGGTCCCTAGCAG-3' |
|  | R: 5'-GCGGCTACATGCACACCTGAGAA-3' |
| NLRP3 | F: 5'-GTGGAGATCCTACGTTTCTCTG-3' |
|  | R: 5'-CAGGATCTCATTCTCTTGGATC-3' |
| GAPDH | F: 5'-TTCAACGGCACAGTCAAGG-3' |
|  | R: 5‘-CTCAGCACCAGCATCACC-3’ |
| TNF-α | F: 5'-CCAGGAGAAAGTCAGCCTCCT-3' |
|  | R: 5'-TCATACCAGGGCTTGAGCTCA-3' |
| TGF-β | F: 5'-CCATGACATGAACCGACCCT-3' |
|  | R: 5'-CCGGGTTGTGTTGGTTGTAG-3' |
| IL-1β | F: 5'-TGCTGTCTGACCCATGTGAG-3' |
|  | R: 5'-GTCGTTGCTTGTCTCTCCTTG-3' |
| IL-10 | F: 5'-AGTGGAGCAGGTGAAGAATG-3' |
|  | R: 5'-CCAGCCTTAGGATCGAAGTT-3' |
| Occludin | F: 5'- TCTTTGTATAAGTCACCGCCT-3' |
|  | R: 5'-CCAGCCTTAGGATCGAAGTT-3' |
| Claudin-1 | F: 5'-ATCGTTCCTAATAAGAACAGAGCC-3' |
|  | R: 5'-GAAGGTGTCTCTAGGTTATCGT-3' |
