# Supplementary figures and images for: Ursolic acid reverses liver fibrosis by inhibiting NOX4/NLRP3 inflammasome pathways and bacterial dysbiosis
Source: Gut Microbes. 2021 Sep 16;13(1):1972746. doi: 10.1080/19490976.2021.1972746 (PMC8451456; doi:10.1080/19490976.2021.1972746)

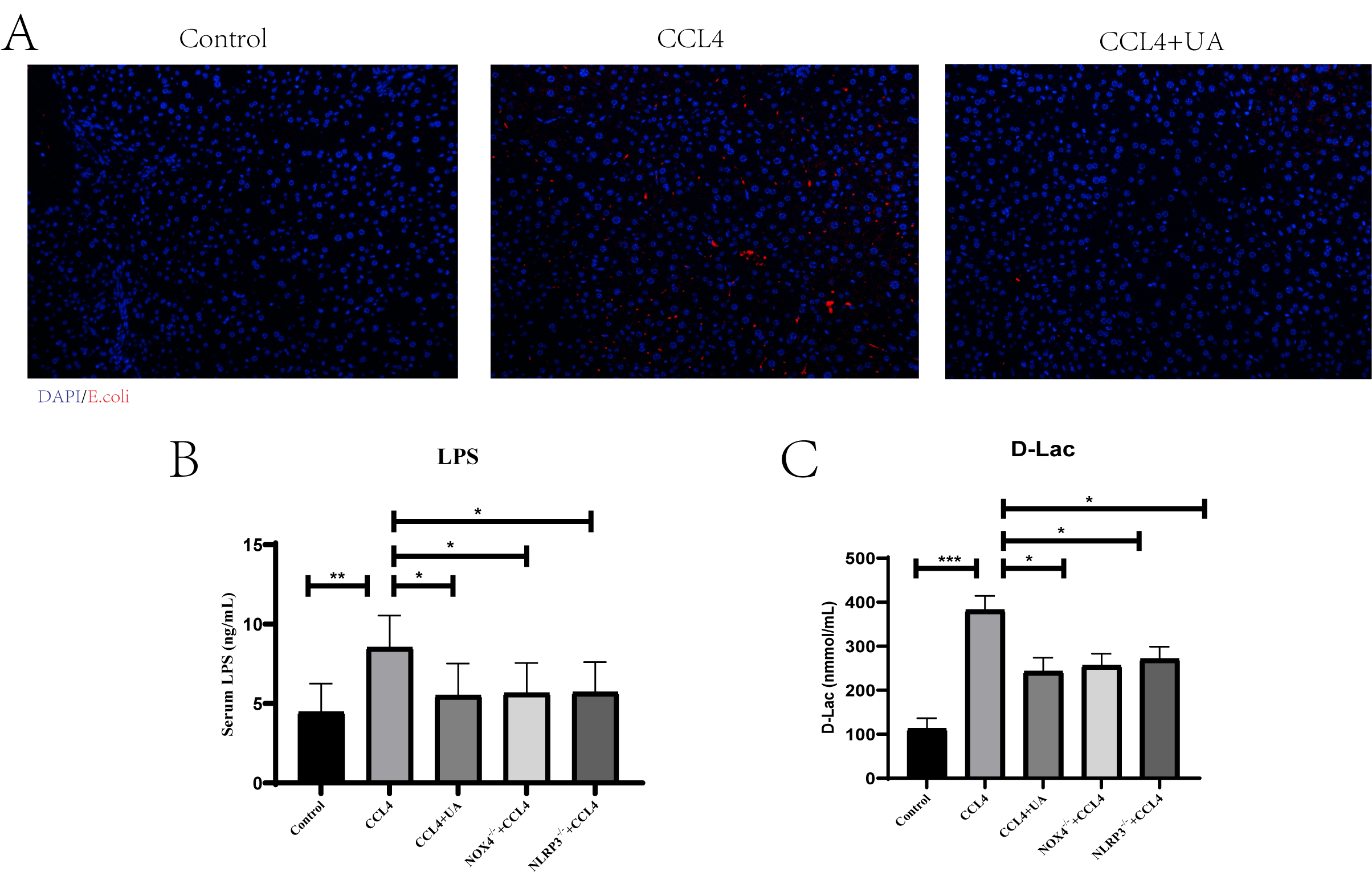

Supplement: Supplemental Material [file KGMI_A_1972746_SM9264.zip › Supplementary information/Supplementary Figure 1.tif]

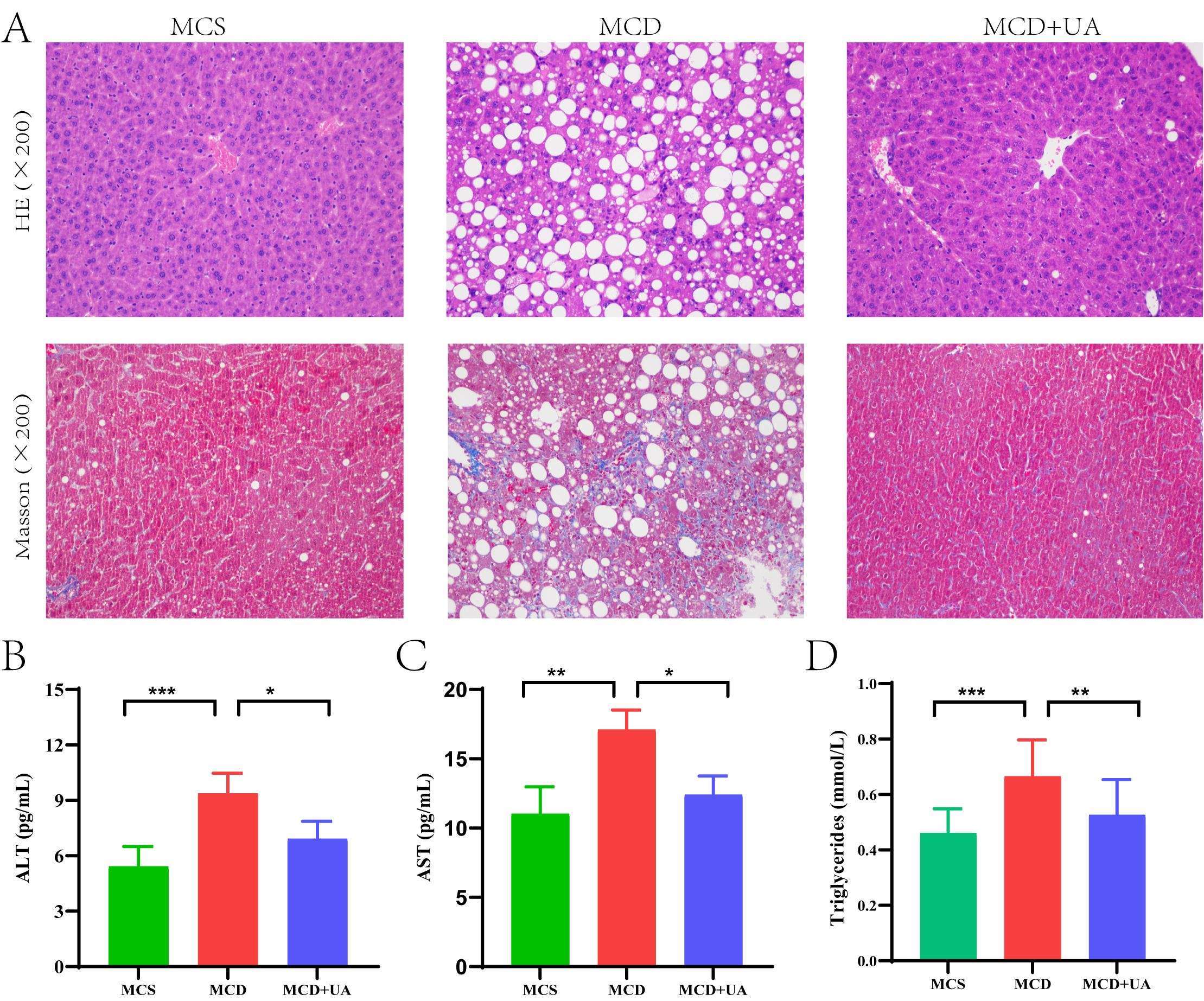

Supplement: Supplemental Material [file KGMI_A_1972746_SM9264.zip › Supplementary information/Supplementary Figure 2.tif]

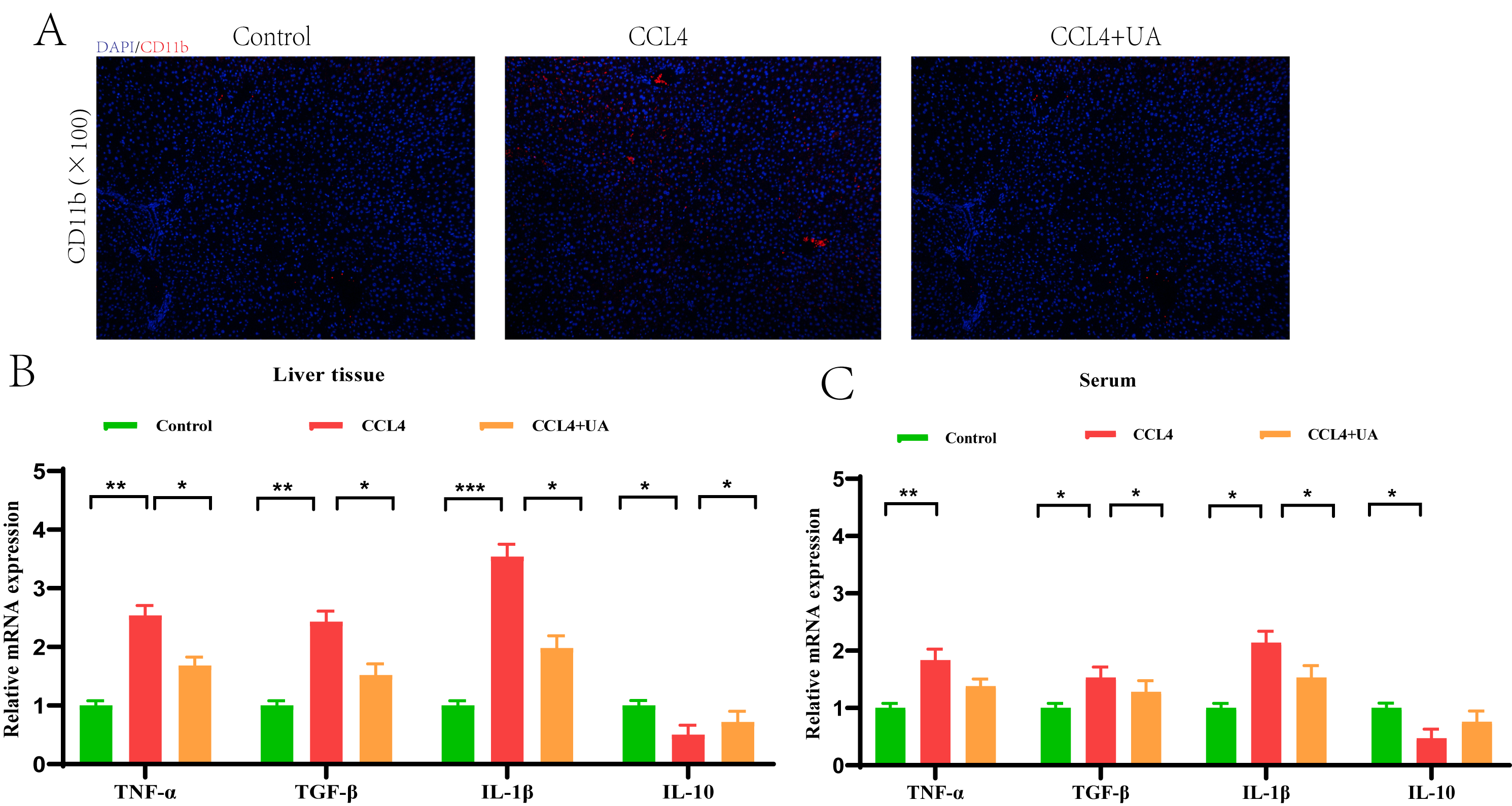

Supplement: Supplemental Material [file KGMI_A_1972746_SM9264.zip › Supplementary information/Supplementary Figure 3.tif]

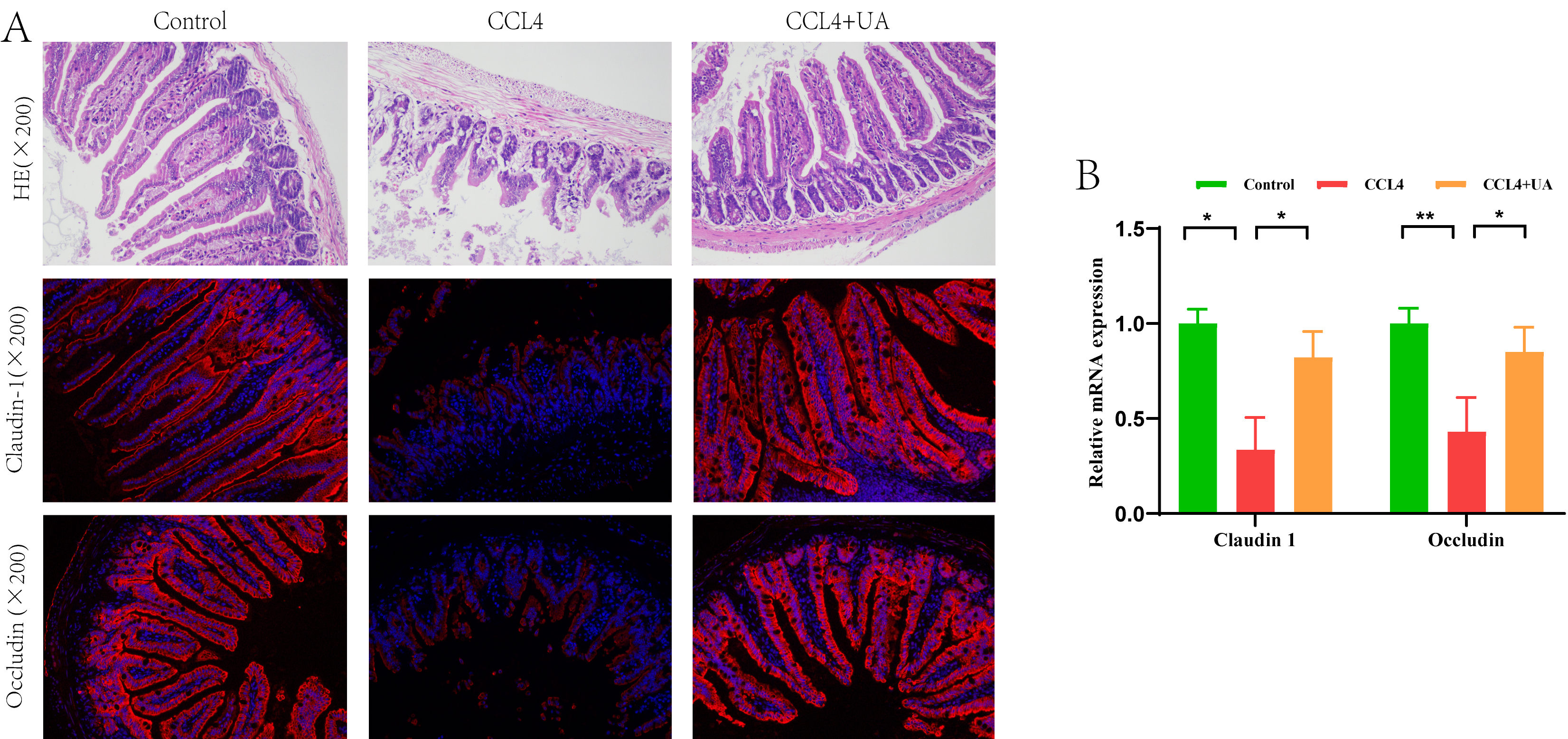

Supplement: Supplemental Material [file KGMI_A_1972746_SM9264.zip › Supplementary information/Supplementary Figure 4.tif]
